# Computer-Aided Design and Computer-Aided Manufacturing (CAD-CAM)-Assisted Telescopic Overdenture for a Post-mucormycosis Maxillary Defect: A Case Report

**DOI:** 10.7759/cureus.113989

**Published:** 2026-08-05

**Authors:** Gaurang Mistry, Sheetal Parab, Sanket Deolekar, Ishita Dua, Ashmita Chabria, Sanpreet S Sachdev

**Affiliations:** 1 Prosthodontics, DY Patil University School of Dentistry, Navi Mumbai, IND; 2 Oral Pathology and Microbiology, Bharati Vidyapeeth (Deemed to Be University) Dental College and Hospital, Navi Mumbai, IND

**Keywords:** cad/cam, maxillary defect, mucormycosis, overdenture, telescopic denture

## Abstract

Post-COVID-19 mucormycosis may result in acquired maxillary defects that compromise mastication, speech, facial support, and separation between the oral cavity and sinonasal spaces. Rehabilitation becomes particularly challenging when the defect is associated with persistent oroantral communication and nasal regurgitation. This clinical report describes the prosthodontic rehabilitation of a 66-year-old man with an Aramany Class II right posterior maxillary defect following mucormycosis. Teeth 11, 12, and 17 were selected as strategic abutments. A staged approach was followed, beginning with a transitional prosthesis with defect extension and soft lining during healing. After tissue stabilization, a definitive telescopic overdenture with a solid obturator extension was fabricated. The primary conical copings were digitally designed using intraoral scanning and computer-aided design software, while the secondary copings were fabricated conventionally over the primary copings and incorporated into the denture base. Functional border molding and a zinc oxide eugenol wash impression were used to record the defect. The prosthesis was processed using heat-polymerized acrylic resin and evaluated at 24 hours, one week, one month, and three months. Nasal regurgitation resolved completely, and the prosthesis remained clinically stable during short-term follow-up. This case demonstrates a conservative tooth-supported approach for selected post-mucormycosis maxillary defects.

## Introduction

Mucormycosis is an aggressive opportunistic fungal infection caused by fungi of the order Mucorales, and its angioinvasive nature may lead to thrombosis, tissue necrosis, and rapid destruction of involved craniofacial tissues [1]. During the COVID-19 pandemic, a marked increase in rhino-orbito-cerebral and rhino-maxillary mucormycosis was reported, particularly in patients with diabetes, corticosteroid exposure, and immune dysregulation associated with SARS-CoV-2 infection [2]. Management of rhinomaxillary mucormycosis commonly requires early antifungal therapy with surgical debridement or resection of necrotic tissues to control disease progression [1]. Although such intervention is often necessary for infection control, it may result in acquired maxillary defects that compromise the separation between the oral cavity and the nasal or maxillary sinus spaces [3].

Acquired maxillary defects can affect speech, mastication, swallowing, facial support, and overall oral function [4]. Persistent oroantral or oronasal communication may lead to nasal regurgitation, altered resonance, difficulty in chewing, compromised esthetics, and reduced social confidence [3]. The primary objective of prosthodontic rehabilitation is therefore to restore the lost oral-sinonasal barrier while providing a prosthesis that is stable, retentive, hygienic, comfortable, and acceptable for long-term use [4].

Several treatment options are available for rehabilitation of acquired maxillary defects, including conventional obturator prostheses, cast partial denture designs, hollow-bulb obturators, implant-supported obturators, and surgical reconstruction [3]. The selection of a prosthetic option depends on the extent and location of the defect, quality of the remaining tissues, number and distribution of residual teeth, systemic condition of the patient, surgical prognosis, cost, hygiene maintenance, and patient preference. In partially dentate maxillary defects, prosthetic design is strongly influenced by the position and periodontal condition of the remaining teeth [5]. The Aramany classification remains a useful guide for planning obturator prostheses in partially edentulous maxillectomy patients, with Class II defects requiring particular attention to cross-arch stabilization, indirect retention, and control of prosthesis rotation toward the defect [5].

Telescopic overdentures provide an alternative retentive approach when strategic abutment teeth are available. They consist of primary copings cemented to prepared abutments and secondary copings incorporated into the removable prosthesis, thereby improving frictional retention, path-of-insertion control, support, retrievability, and hygiene access [6]. Compared with conventional clasp-retained obturators, telescopic designs may improve esthetics and reduce visible retentive components while using the remaining teeth for prosthetic control. Digital scanning and Computer-Aided Design and Computer-Aided Manufacturing (CAD-CAM) workflows can further assist in evaluating restorative space, designing a common path of insertion, standardizing coping morphology, and improving communication between the clinician and laboratory during fabrication [7]. This clinical report describes the rehabilitation of a post-mucormycosis Aramany Class II maxillary defect using a CAD-CAM-assisted telescopic overdenture with obturator extension.

## Case presentation

A 66-year-old man presented to the institutional Department of Prosthodontics with the chief complaint of missing maxillary teeth, difficulty masticating, and nasal regurgitation of food and fluids. He had a history of COVID-19-associated mucormycosis involving the right posterior maxillary region. The diagnosis of mucormycosis had been confirmed from the patient’s previous medical records, and the patient was systemically stable at the time of prosthetic rehabilitation. The patient received intravenous liposomal amphotericin B therapy for three weeks and underwent two surgical debridement/curettage procedures for removal of the affected tissues. Despite surgical management, a residual buccal defect with an oroantral fistula persisted, resulting in continued nasal regurgitation. Prosthetic rehabilitation was initiated four weeks after the final surgical debridement, once clinically satisfactory healing and tissue stability had been achieved. The residual defect was classified as an acquired Aramany Class II maxillary defect.

Clinical examination

Intraoral examination revealed a partially edentulous maxillary arch with an acquired right posterior maxillary defect and persistent oroantral communication. The remaining maxillary teeth were 11, 12, 16, 17, 21, 22, 23, 24, and 27. The opposing mandibular arch consisted of natural dentition extending from 37 to 47. Teeth 11, 12, and 17 were selected as abutments for the definitive telescopic prosthesis based on their clinical condition, radiographic findings, and strategic distribution. Teeth 11 and 12 were periodontally sound, showed no clinical mobility, and were asymptomatic, with no signs suggestive of pulpal or periapical pathology. Radiographic examination demonstrated satisfactory alveolar bone support around teeth 11 and 12, with an intact lamina dura and no evidence of periapical pathology. Tooth 17 exhibited Miller Class III gingival recession with an exposed root surface but showed no clinical mobility or symptoms [8]. Radiographically, 17 showed adequate remaining alveolar bone support without periapical pathology or other findings that contraindicated its use as an abutment. Although the periodontal prognosis of 17 was considered fair because of the gingival recession, it was retained as the distalmost available abutment adjacent to the defect. Tooth 17 was preferred over 16 because its more distal position provided a wider anteroposterior distribution of support and improved control of prosthesis rotation toward the defect. The exposed root surface of 17 was not completely covered by the coping to avoid excessive contouring, facilitate plaque control, and maintain access for oral hygiene.

Treatment planning

The treatment objectives were to obturate the residual oroantral communication, control nasal regurgitation, restore mastication and speech, provide adequate facial support, and obtain a retentive and stable removable prosthesis. A staged prosthodontic approach was selected. A transitional prosthesis was first used during the healing period, followed by definitive rehabilitation with a tooth-supported telescopic overdenture incorporating a solid obturator extension.

Conical/tapered primary and secondary metal copings were planned to provide frictional retention and a defined path of insertion. Full palatal coverage was incorporated to provide broad tissue support and cross-arch stabilization. As the prosthesis was an overdenture rather than a conventional cast partial denture, additional clasp retention on the contralateral side was not considered necessary. The 26 region was restored as part of the definitive tooth arrangement to complete the contralateral occlusal table and contribute to functional cross-arch support.

Transitional prosthetic phase

**Figure 1:**
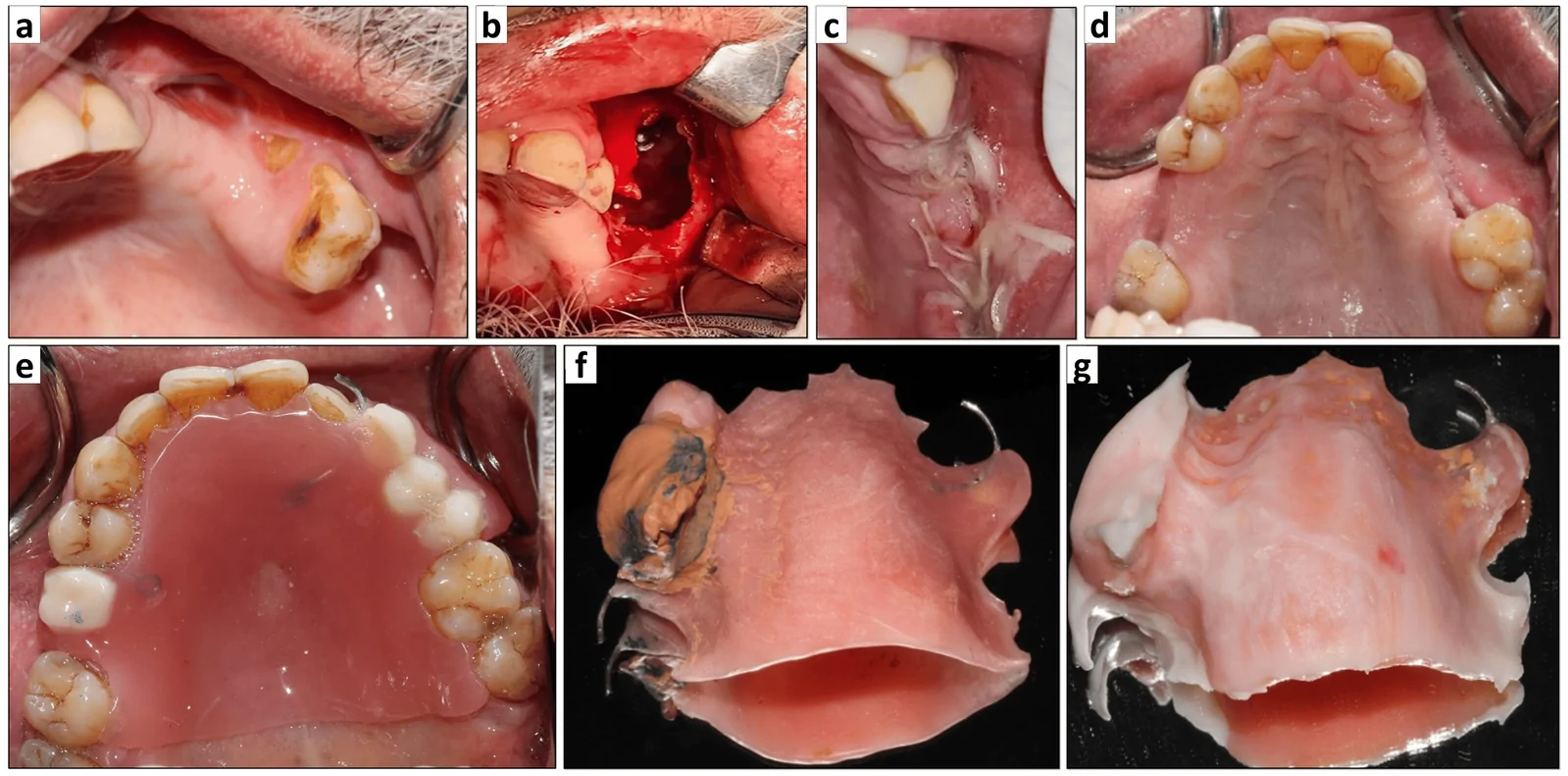
(a) Buccal view of the right posterior maxillary defect. (b) Surgical debridement/curettage of the fistulous region. (c) Postoperative healed surgical site with persistent defect. (d) Preoperative intraoral view of the maxillary arch. (e) Transitional prosthesis in position. (f) Border molding of the defect region for the transitional prosthesis. (g) Long-term soft liner applied to the tissue surface of the transitional prosthesis

Following surgical debridement and initial healing, a transitional removable prosthesis with a bulb extension was fabricated to obturate the residual defect. The tissue-contacting surface was relined with a long-term soft liner (Mollosil; DETAX GmbH & Co. KG, Ettlingen, Germany) to minimize direct contact of the acrylic resin with the healing tissues and permit adaptation to ongoing tissue changes. The transitional phase allowed progressive functional adaptation while the surgical site matured before definitive rehabilitation. The defect, surgical curettage, subsequent healing, transitional prosthesis, border molding, and soft-liner application are illustrated in Figure 1.

Definitive prosthetic phase

After stabilization of the surgical tissues, definitive prosthetic rehabilitation was initiated. An arbitrary centric jaw relation was recorded using conventional occlusal rims against the natural mandibular dentition. The existing vertical dimension of occlusion was considered clinically acceptable and was therefore maintained without alteration. A bilateral balanced occlusal scheme was planned to distribute functional contacts and reduce destabilizing forces and rotation toward the defect side.

**Figure 2:**
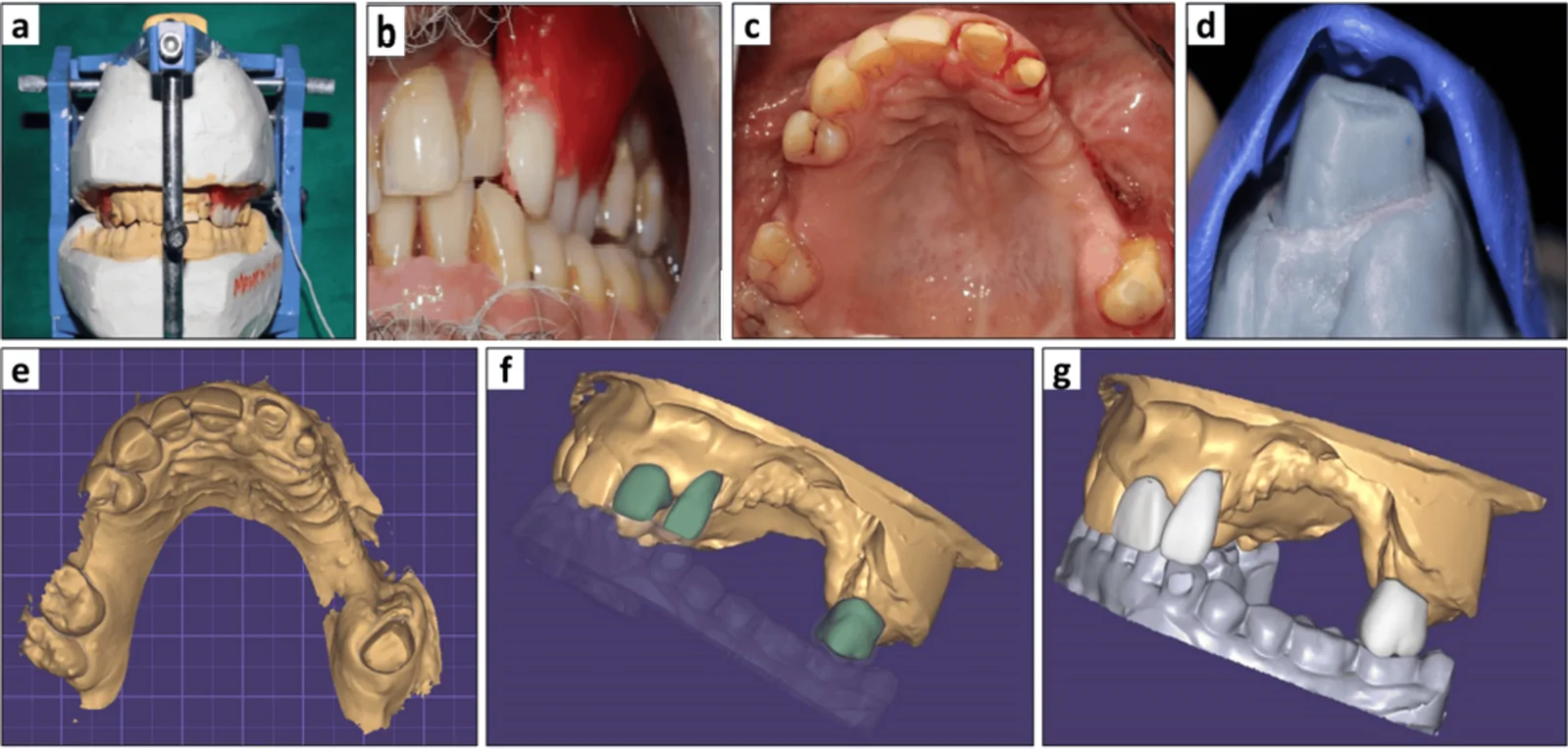
(a) Tentative tooth arrangement. (b) Wax trial. (c) Prepared abutment teeth 11, 12, and 17. (d) Putty index used to evaluate clearance for the telescopic coping assembly. (e) Digital scan of the prepared abutments. (f) CAD design of the primary copings. (g) Conventionally fabricated secondary copings before incorporation into the definitive prosthesis CAD: computer-aided design

A tentative tooth arrangement was completed and evaluated at the wax trial. The trial arrangement was subsequently used to fabricate a putty index (Zetaplus; Zhermack SpA, Badia Polesine, Rovigo, Italy) to assess the available restorative space and clearance required for the telescopic coping assembly. Teeth 11, 12, and 17 were then prepared to receive conical/tapered primary copings. The definitive tooth arrangement, wax trial, abutment preparation, putty index, digital scan, primary coping design, and conventionally fabricated secondary copings are shown in Figure 2.

Digital workflow and coping fabrication

Following tooth preparation, an intraoral digital scan was obtained using a 3Shape TRIOS intraoral scanner (3Shape A/S, Copenhagen, Denmark). The primary copings were digitally designed using the exocad DentalCAD software (exocad GmbH, Darmstadt, Germany). The digital workflow facilitated visualization of the available restorative space, establishment of a common path of insertion, and control of the conical/tapered configuration of the primary copings. The primary copings were fabricated from cobalt-chromium alloy (Ceramill Sintron; Amann Girrbach AG, Koblach, Austria) and clinically evaluated for complete seating, marginal adaptation, and common path of insertion. After satisfactory verification, they were cemented onto teeth 11, 12, and 17 using glass ionomer cement (GC Fuji I; GC Corporation, Tokyo, Japan).

The secondary copings were fabricated conventionally rather than through the CAD-CAM workflow. With the primary copings seated intraorally, an impression was made using a medium-body elastomeric impression material in a custom tray to reproduce their position and orientation. The secondary cobalt-chromium copings were conventionally fabricated over the corresponding primary copings and were incorporated into the heat-polymerized acrylic denture base during processing. No additional metal framework or reinforcement was incorporated. Their intimate adaptation to the primary copings provided the frictional retention required for the telescopic overdenture.

Definitive impression and prosthesis fabrication

With the secondary copings in position, functional border molding of the defect was performed using low-fusing thermoplastic impression compound (DPI Pinnacle Tracing Sticks; Dental Products of India Ltd., Mumbai, India). No major clinically relevant undercuts were present within the defect; therefore, extensive block-out was not required. Care was taken during impression making and retrieval to prevent undue extension of the impression material into the oroantral communication. Since no major undercuts were present, no extensive block-out was required. The impression material was restricted to the functionally recorded defect boundaries to avoid displacement into the oroantral communication. A wash impression was subsequently made using zinc oxide eugenol impression paste to record the supporting tissues and functional borders of the defect. The impression was carefully retrieved along the planned path of removal, inspected for complete recording of the defect margins and absence of retained impression material, and poured using Type IV dental stone (Elite Rock; Zhermack SpA, Badia Polesine, Rovigo, Italy) to obtain the master cast.

**Figure 3:**
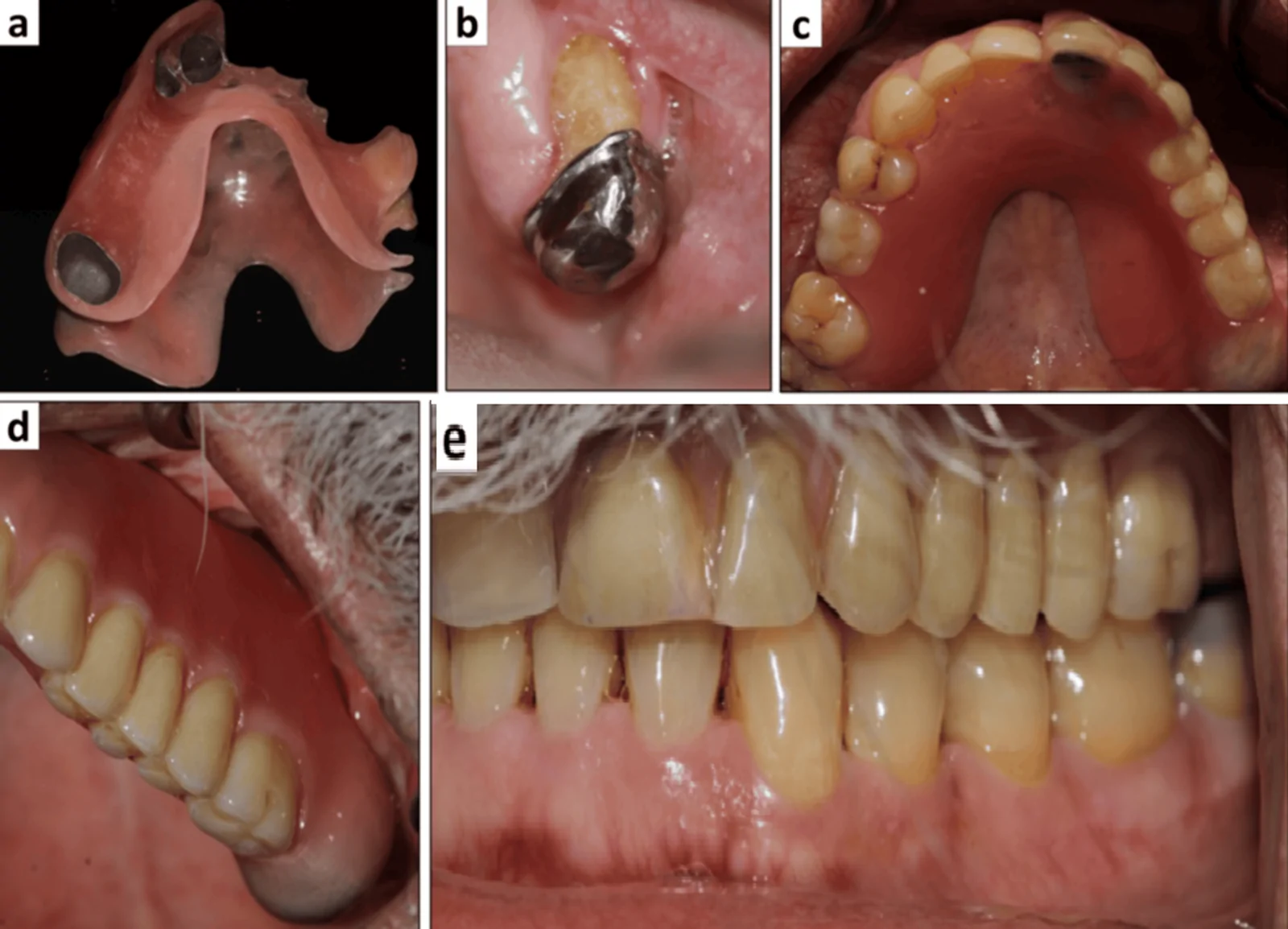
(a) Definitive telescopic overdenture showing the secondary coping components. (b) Cemented primary copings on the selected abutments. (c) Definitive overdenture in position. (d) Solid obturator extension occupying the right posterior buccal defect. (e) Postoperative intraoral view of the definitive prosthesis in occlusion

The obturator component was designed as a solid extension and was limited to the functionally recorded boundaries of the buccal defect to obtain closure without unnecessary tissue impingement. Its external surface remained accessible for routine cleaning after removal of the prosthesis. The definitive overdenture was processed using heat-polymerized polymethyl methacrylate resin (Trevalon HI; Dentsply India Pvt. Ltd., Gurugram, India) by the conventional compression molding technique. Acrylic resin teeth (SR Orthotyp DCL; Ivoclar Vivadent AG, Schaan, Liechtenstein) were arranged according to the planned bilateral balanced occlusal scheme. The final telescopic overdenture, primary and secondary coping relationship, defect extension, and intraoral occlusion are presented in Figure 3.

Prosthesis delivery

The definitive prosthesis was evaluated intraorally for seating, retention, stability, occlusion, extension into the defect, and patient comfort. Retention was derived predominantly from frictional engagement between the primary and secondary copings, while full palatal coverage provided broad tissue support and cross-arch stabilization. Restoration of the 26 region completed the contralateral occlusal table and contributed to functional support across the arch. The bilateral balanced occlusal contacts were verified against the natural mandibular dentition and adjusted where required to minimize destabilizing forces.

Retention and stability were assessed clinically during insertion, removal, and functional mandibular movements. Pressure areas were identified and relieved as required. The patient was instructed regarding insertion and removal of the prosthesis, cleaning of the primary copings and exposed root surfaces, hygiene of the solid obturator extension, and the importance of regular recall examinations.

Follow-up and outcome

The patient was recalled at 24 hours, one week, one month, and three months after delivery. At the 24-hour appointment, minor pressure areas were adjusted. At the one-week and one-month visits, the patient reported progressive improvement in comfort, speech, and mastication. The patient also reported complete resolution of nasal regurgitation following definitive prosthesis delivery. Clinically, the prosthesis showed satisfactory retention and stability during functional assessment, while the periabutment tissues and margins of the defect were inspected at each recall. At the three-month follow-up, the periabutment tissues showed no clinically evident inflammation, ulceration, tissue impingement, or other adverse soft-tissue response, and no major prosthetic complication was observed. These findings represent short-term clinical and patient-reported outcomes and were not based on quantitative assessment of retention, masticatory efficiency, speech, or oral health-related quality of life.

## Discussion

Rehabilitation of an acquired unilateral posterior maxillary defect following mucormycosis presents a biomechanical and functional challenge because the resulting impairment extends beyond replacement of missing teeth. Oroantral communication, loss of posterior support, altered tissue contours, and reduced support on the defect side can predispose a removable prosthesis to displacement and rotation during function. In the present case, these concerns were addressed by combining a solid obturator extension with a tooth-supported telescopic overdenture, thereby obtaining defect closure while using the remaining strategic teeth and palatal tissues for additional prosthetic support.

Maxillary defects following surgical management can adversely affect mastication, swallowing, speech, facial contour, and social functioning. Recent clinical analyses of maxillectomy patients have emphasized the functional and anatomical consequences of loss of maxillary structures and the importance of appropriate reconstructive or prosthetic rehabilitation [9]. In patients treated for mucormycosis, repeated debridement may additionally produce irregular tissue contours and a changing surgical bed. Delaying definitive rehabilitation until infection control and tissue stabilization had been achieved was therefore important in the present case. The transitional prosthesis permitted early functional adaptation, while the definitive prosthesis was fabricated only after maturation of the surgical tissues.

Conventional obturators, hollow-bulb designs, and cast partial dentures have been widely employed for acquired maxillary defects to reestablish separation between the oral and sinonasal spaces [10]. However, their retention may depend considerably on defect undercuts, residual teeth, clasps, and available tissue support. The present design used conical primary and secondary copings to obtain frictional retention from the remaining abutments. This reduced reliance on the defect itself as a retentive area and provided a defined path of insertion for the overdenture.

Selection and distribution of the abutments were particularly important for controlling prosthesis movement. Teeth 11 and 12 provided anterior support, while 17, despite its fair periodontal prognosis, was preserved because its distal position increased the anteroposterior spread of support and placed an abutment closer to the defect. Full palatal coverage provided broad tissue support and cross-arch stabilization. Replacement of the 26 region completed the contralateral occlusal table and contributed to functional support on the nondefect side. As the prosthesis functioned as a telescopic overdenture rather than a conventional cast partial denture, additional clasp retention was not incorporated.

Telescopic overdentures offer frictional retention, a controlled path of insertion, retrievability, splinting of selected abutments, and favorable esthetics. Clinical investigations of double-crown and telescopic removable prostheses have demonstrated satisfactory retention and patient acceptance, although long-term success depends on preservation of abutment health, maintenance of coping friction, and adequate oral hygiene [11,12]. These features were useful in the present defect situation, where limiting rotational movement was important for maintaining the peripheral seal and minimizing tissue irritation.

The staged use of an interim prosthesis was also advantageous. Transitional obturation can restore basic function while accommodating postoperative tissue changes before definitive rehabilitation. Similar approaches have been advocated for acquired maxillary defects, where continued tissue remodeling may otherwise compromise the adaptation of an early definitive prosthesis [10]. The use of a soft liner additionally provided a more accommodating tissue surface during this healing period.

The digital workflow facilitated fabrication of the primary coping assembly. Intraoral scanning and CAD design allowed visualization of restorative space, establishment of a common path of insertion, and controlled design of the conical primary copings. Digital data also simplified communication between the clinical and laboratory stages and provided a reproducible reference for coping morphology, consistent with the broader advantages reported for digital workflows in maxillofacial and restorative dentistry [13]. The secondary copings were subsequently fabricated conventionally over the primary copings, allowing the digital and conventional stages to be combined according to the requirements of the prosthesis.

An implant-supported obturator was another possible approach but would have required additional surgical intervention and adequate residual bone. Implant-assisted rehabilitation may offer enhanced retention in selected patients, but posterior maxillary bone availability, sinus anatomy, surgical morbidity, systemic factors, and patient preference may influence its suitability [14]. Preservation of the available teeth therefore allowed a less invasive definitive rehabilitation in this patient.

Complete resolution of nasal regurgitation after delivery represented the most relevant patient-reported functional outcome and suggested effective obturation of the persistent oroantral communication. Nevertheless, the outcome should be interpreted within the limitations of a single case with only three months of follow-up. Retention force, coping fit, masticatory efficiency, speech, and oral health-related quality of life were not objectively quantified. Longer follow-up is required to assess periodontal health of the abutments, maintenance of telescopic friction, cement integrity, tissue response, and prosthesis durability.

## Conclusions

A CAD-CAM-assisted telescopic overdenture with a solid obturator extension provided a stable removable prosthetic option for this post-mucormycosis Aramany Class II maxillary defect with persistent oroantral communication. The use of strategic abutments together with full palatal coverage contributed to retention and cross-arch stabilization, with complete resolution of nasal regurgitation during the three-month follow-up. Longer follow-up is required to assess maintenance of abutment health, telescopic retention, and prosthesis durability.
